# The role of anion gap normalization time in the management of pediatric diabetic ketoacidosis

**DOI:** 10.3389/fped.2023.1198581

**Published:** 2023-05-30

**Authors:** Isaac Lazar, Dorit Wizeman-Orlov, Guy Hazan, Asaf Orbach, Alon Haim, Yuval Cavari, Yael Feinstein, Eitan Neeman, Eli Hershkovitz, Yaniv Faingelernt

**Affiliations:** ^1^ Pediatric Intensive Care Unit, Saban Center of Pediatrics, Soroka University Medical Center, Beer Sheva, Israel; ^2^ Faculty of Health Sciences, Ben-Gurion University of the Negev, Beer Sheva, Israel; ^3^ Department of Pediatrics D, Saban Center of Pediatrics, Soroka University Medical Center, Beer Sheva, Israel; ^4^ Pediatric Endocrinology Unit, Saban Center of Pediatrics, Soroka University Medical Center, Beer Sheva, Israel

**Keywords:** diabetic ketoacidosis (DKA) - in children or adolescents with established type 1 diabetes, anion gap (AG), pediatric intensive care unit (PICU), children, type 1 diabetes mellitus (DM1), anion gap normalization time, DKA resolution

## Abstract

**Introduction:**

Our aims were to determine whether anion gap normalization time (AGNT) correlates with risk factors related to the severity of diabetic ketoacidosis (DKA) in children, and to characterize AGNT as a criterion for DKA resolution in children admitted with moderate or severe disease.

**Methods:**

A ten-year retrospective cohort study of children admitted to the intensive care unit with DKA. We used a survival analysis approach to determine changes in serum glucose, bicarbonate, pH, and anion gap following admission. Using multivariate analysis, we examined associations between patients' demographic and laboratory characteristics with delayed normalization of the anion gap.

**Results:**

A total of 95 patients were analyzed. The median AGNT was 8 h. Delayed AGNT (>8 h) correlated with pH < 7.1 and serum glucose >500 mg/dL. In multivariate analysis, glucose >500 mg/dL was associated with an increased risk for delayed AGNT, by 3.41 fold. Each 25 mg/dL elevation in glucose was associated with a 10% increment in risk for delayed AGNT. Median AGNT preceded median PICU discharge by 15 h (8 vs. 23 h).

**Discussion:**

AGNT represents a return to normal glucose-based physiology and an improvement in dehydration. The correlation observed between delayed AGNT and markers of DKA severity supports the usefulness of AGNT for assessing DKA recovery.

## Introduction

Diabetic ketoacidosis (DKA) is a common complication in children diagnosed with type 1 diabetes mellitus (1, 2). The DKA syndrome includes hyperglycemia, metabolic acidosis, glycosuria, and severe dehydration (3). Relative or absolute lack of insulin causes a shift from mainly glucose-based energy production to almost exclusive fatty acid utilization (2, 4). As end products of fatty acids metabolism, organic acids, mainly ketone bodies (KB) (5) are produced and accumulate in the serum, causing high anion gap metabolic acidosis (4, 6). High serum glucose causes forced diuresis, together with nausea and vomiting due to cytokine storm worsens dehydration. Severe dehydration may interfere with KB elimination due to poor perfusion to skeletal muscles and the kidneys (which may intensify the acidosis) but also imply poor skin perfusion and decreased subcutaneous insulin absorption and demand for IV insulin treatment (3, 4, 7). Although varied by institution, pediatric patients with moderate to severe DKA are admitted to a highly specialized unit—the pediatric intensive care unit (PICU) or other similar units such as endocrinology special unit, enabling close monitoring and professional clinical care (1, 3). The rationales behind these recommendations are the complexity of treatment and the close follow-up these patients deserve. Once the DKA crisis resolves, treatment can be de-intensified, and patients are transferred to the general ward (3). DKA is considered resolved once acidosis is corrected, ketosis is reversed (KB production is stopped and cleared from the circulation), dehydration is corrected, serum glucose is close to normal, and no complications occurred from the acute event or the treatment (2, 3). Different publications attempted to better define a biochemical marker/s for DKA resolution (8–12). Serum pH, bicarbonate, serum or urine KB, and serum anion gap (AG) were all studied. During the initial course of DKA admission, metabolic acidosis is caused mainly by high serum KB and other organic acids, with normal serum chloride, as reflected by wide anion gap metabolic acidosis (4, 5, 10). However, as treatment continues, KB production ceases and improved skeletal muscles and kidney (and other organs) (3, 4, 7) perfusion facilitates KB clearance from the serum. Concomitantly, in many described patients (10–12), serum chloride levels increase, causing normal anion gap metabolic acidosis, which usually persists for hours and sometimes days despite the resolution of DKA (3, 10, 13). Hyperchloremic metabolic acidosis (HMA) carries a low risk of complications and its management with rehydration is a common practice on the pediatric medical floors. We suggest that anion gap serum values have an important role in the understanding of DKA pathophysiology and that anion gap normalization indicates DKA resolution. We thought that timely changes in pH, bicarbonate, sodium, chloride, and other biochemical characteristics, together with epidemiological, socioeconomic, and ethnic conditions (3, 14–16) may provide insight into the DKA process and its resolution. We hypothesized that the anion gap normalization time (AGNT) may correlate with risk factors for severe DKA, and with markers of DKA severity. We also hypothesized that AGNT may be a good surrogate marker for DKA resolution. Our objectives were to characterize AGNT in patients admitted to the PICU with DKA. The importance of our study is that maintaining intravenous insulin infusion, frequent blood sampling, and close monitoring in the PICU beyond AGNT and until pH/bicarbonate normalizes may be unnecessary.

## Methods

Setting: The study was conducted at Soroka University Medical Center, the only tertiary medical hospital in southern Israel, which encompasses about two-thirds of the country's geographical area. It is the main medical care facility for over 1.1 million inhabitants and an estimated pediatric population of about 400,000 infants and children (17). The study was approved by the local ethical committee (# SOR-180-0269).

Patients: All patients presented to the medical center with moderate to severe DKA were admitted to PICU and treated according to a similar designated treatment protocol (1, 3), in collaboration with the endocrinology service. In brief, our PICU DKA protocol, which is derived from the ISPAD guidelines, admits all children with moderate to severe DKA to our unit. Two peripheral IV lines and an arterial line are placed. Continuous infusion of regular insulin is infused at a rate of 0.1 unit\Kg\hour. IV fluids (saline 0.9% or saline 0.45%) are given at an infusion rate according to the level of dehydration the patient arrived with, for as long as serum glucose is measured above 300 mg\dL. Once dropped <300 mg\dL, dextrose is added to the IV solutions to maintain serum levels of 100–200 mg\dL. Once pH rises above 7.30 and bicarbonate >18 mEq/L and the patient's general condition has improved, the patient is transferred to the general pediatric floor with intermittent subcutaneous (SC) insulin injections under pediatric endocrinologist guidance.

The study inclusion criteria were age under 18 years, a diagnosis of pediatric DKA (blood glucose >200 mg/dL and blood pH less than 7.2, **or** a serum bicarbonate level less than 15 mmol/L), and treatment with intravenous insulin. AG was calculated as serum [Na] – {[bicarbonate] + [chloride]} (normal values 12 ± 2 mEq\L) (4). The study exclusion criteria were the lack of electronic medical records, such as due to technical problems, ketoacidosis secondary to non-diabetic causes, the initiation of treatment for DKA in another hospital or department in our center, and the absence of data to calculate a valid AG or AGNT.

Data collection and definitions of variables: Patient data were extracted from the PICU electronic medical record system (Metavision. IMD Soft. Inc. Boston, MA. USA), which was fully implemented from the beginning of 2008. A designated database was built on a Microsoft Excel (Ver. 2010, Microsoft, VA. USA) spreadsheet. Demographic data were extracted from admission reports. Vital signs on admission were defined as those taken in the PICU. Laboratory findings on admission were defined as the first laboratory findings taken on admission to the hospital (the emergency department or the PICU). Levels of pH (normal 7.35–7.45), pCO2 (normal 35–45 torr), and bicarbonate (normal 22–26 mEq/L) were based on blood gas analysis (venous or arterial). Levels of sodium, potassium, chloride, and all other electrolytes are based on serum chemistry lab analysis. Blood gas samples (analyzed in the PICU) and blood chemistry samples (analyzed in the hospital's laboratory) were drawn simultaneously. A technical time lag existed between the ABG results and the chemistry laboratory results that were published and recorded in the EMR (time for the blood sample to reach the laboratory, time to be analyzed and published). We decided that a time difference of two hours or less, between the bicarbonate and serum electrolytes levels results recording was considered valid for AG calculation. Results difference recorded over two hours were considered unmatched and non-valid for AG calculation. AG was calculated based on the published formulation of Na serum concentration (mEq/L) minus serum bicarbonate (mEq/L) and serum chloride (mEq/L) concentrations respectively. AG normalization was defined as an anion gap of 12 mEq/L or less, together with normal serum albumin levels (1, 4). We defined the median AGNT (or earlier) as “standard” and AGNT longer than the median as “delayed AGNT”. Data on the admission course, clinical outcomes, complications, and discharge follow-up were all extracted from the hospital's electronic records. Of note is that our study was not aimed to seek the effect of different fluid regimens nor the effect of change of the different fluids given over the admission course.

Statistical analysis: We examined changes in serum glucose, bicarbonate, pH, and AGNT up to 30 h following PICU admission. Demographical, clinical, and laboratory parameters were also analyzed for association with AGNT. The descriptive analysis included analyses of the distribution of single variables, central tendency, and dispersion, in graphical or tabular format. Parameters showing normal population distribution are presented as means and standard deviations. Parameters with scattered distribution are expressed as medians with interquartile range (IQR, 25th to 75th percentiles). Univariate analysis was done by chi-square for dichotomic variables, the Mann-Whitney non-parametric U test for non-parametric continuous variables, the T-test for parametric variables, and the Kaplan-Meier test for survival analysis. The remaining variables (*P*-value < 0.2), other than AGNT, were examined for collinearity by Spearman or Pearson correlation. The strength of the relationship of AGNT and continuous variables were examined for collinearity by the Pearson coefficient and for ordinal data by Spearman's rho coefficient correlations. In multivariate analysis, using logistic regression analysis, we examined associations of demographic and clinical factors with delayed AGNT (as compared to the study population AGNT median value). We conducted multivariate logistic and Cox regression models for variables associated with a *P* value < 0.2 in the univariate analysis. We conducted standard backward elimination by the stepwise logistic regression analysis method. For the multivariate analysis, a *P* value of <0.05 was considered significant. For all independent variables, we ruled out confounding, multi-co-linearity, and interactions. Statistical analysis was conducted using IBM SPSS 21.0 (IBM Corporation, Armonk, New York, USA).

## Results

**Figure 1:**
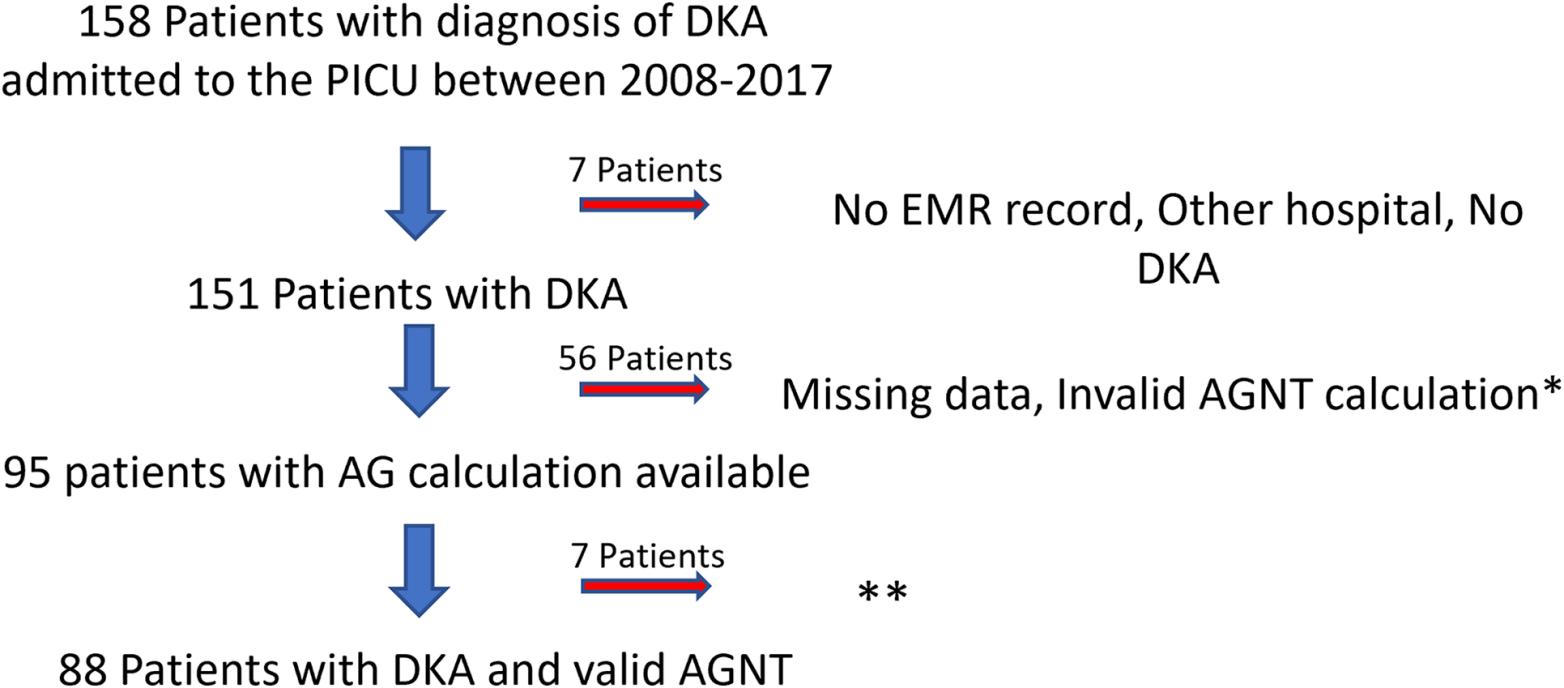
Flow chart of the study population. *Over 2-hour difference between serum chemistry and blood-gas results. **7 patients with AGNT >8hr. DKA, diabetic ketoacidosis; PICU, pediatric intensive care unit; EMR, electronic medical record; AGNT, anion gap normalization time; AG, anion gap.

Participants: Between the 1st of January, 2008, and 31st of December, 2017, 158 patients were admitted with DKA to our PICU. Seven patients were excluded because of a lack of electronic medical record (EMR) (during the early time of the implementation), DKA treatment was initiated in another hospital, or missed diagnosis (non-DKA acidosis). Fifty-six patients were excluded because of the inability to calculate AGNT. These patients' medical charts showed a gap of over two hours between serum electrolyte concentration (for sodium and chloride serum concentration) and blood gas analysis (for bicarbonate serum concentration) laboratory results. Ninety-five met the study inclusion criteria, and their data were available for analysis (Figure 1 flow chart describes patient's inclusion in the study). Although 95 patients met our inclusion criteria, valid data for calculating AGNT was available for only 88. Seven patients with unknown precise AGNT, but who certainly normalized AG over 8 h, were included in some of the analyses (although deviated from the DKA PICU protocol, blood sampling was taken in longer time gaps due to technical or access reasons).

Descriptive data: The mean age at admission was 10.0 ± 4.6 years. Both male-to-female and Jewish-to-Muslim Bedouin distributions were almost equal. Most patients' admission was during their diabetes mellitus diagnosis and first DKA episode (83/95, 87.4%).

**Figure 2:**
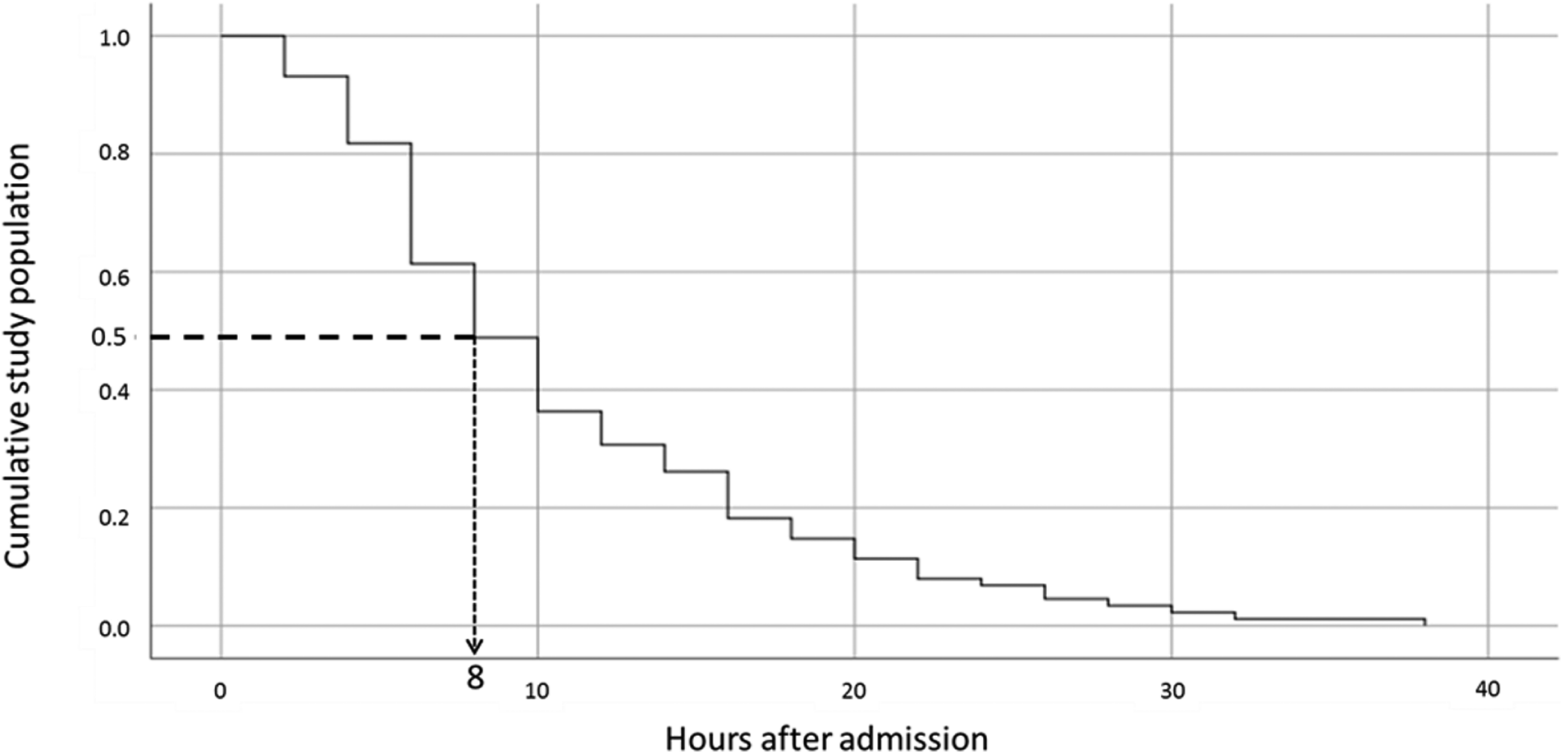
The cumulative median anion gap normalization value according to time since admission. The Kaplan-Meier curve shows the cumulative median anion gap normalization value over time. The dotted line shows that for 50% of the population, the anion gap was normalized by the eighth-hour post-admission.

**Figure 3:**
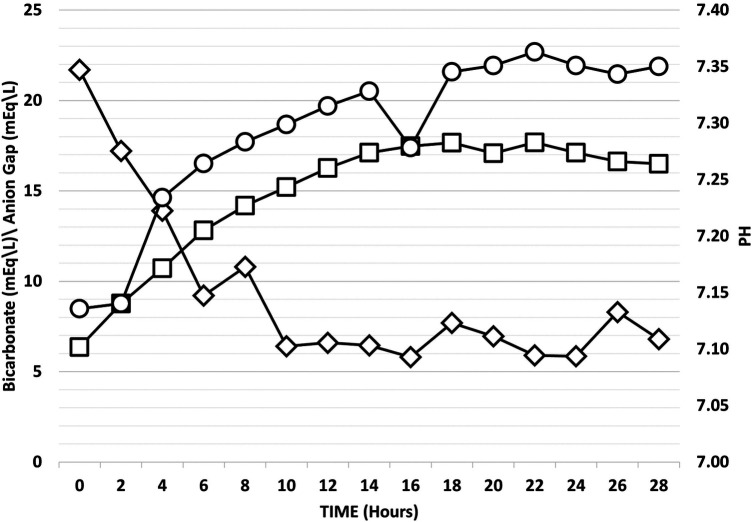
Median pH, bicarbonate, and anion gap values over the first 30 hours following admission. The three curves show changes in parameters in the 30 hours following admission. Diamonds (◊) represent median anion gap values, circles (○) represent median pH levels, and squares (□) represent median bicarbonate levels.

Outcome data: The median PICU length of stay was 23 h (IQR 20–30 h). The mean serum glucose level at presentation was 508 mg/dL and the median admission pH was 7.14 (IQR 7.06–7.19) (Table 1). The median AGNT was 8 h (IQR 6–12 h) after PICU admission (Figure 2). Following steep changes during the first few hours after admission, the AG slopes attenuated at the eighth to tenth hours of admission. pH and bicarbonate levels continue to rise at slower rates, and at the 14th and 16th hours respectively, the slopes flattened (Figure 3). While the mean pH approached normal values (7.35–7.45) the mean serum bicarbonate level did not reach 18 mEq/L even at the 30th hour. Cerebral edema and death were not reported among the study population.

**Table 1:** Demographic and clinical characteristics of the study population.

Demographic/Clinical parameter	Central tendency and dispersion
Age in years (mean ± SD)	10±4.6
Female gender (N, %)	48, 50.5%
Weight in kilograms (mean ± SD)	34.96±17.7
Weight in Z score (mean ± SD)	−0.51±2.22
Muslims-Bedouins (N, %)	49, 51.6%
First episode of DKA (N, %)	83, 87.4%
Glucose level at presentation (mg/dL) (median, IQR)	508 (503-642)
pH level at presentation (median, IQR)	7.14 (7.06-7.19)
PICU length of stay (hours) (median, IQR)	23 (20-30)

DKA, diabetic ketoacidosis; PICU, pediatric intensive care unit; IQR, inter quartile range 25%-75%.

Main results: Upon univariate analysis, admission pH lower than 7.1 was found to be associated with delayed AGNT [odds ratio (OR) 3.15, 95%CI 1.2–8.1, *P* = 0.015] (Table 2). Serum glucose >500 mg/dL on admission was associated with delayed AGNT (>8 h) (OR 2.7, 95%CI 1.2–6.2, *P* = 0.02). Post-pubertal age (>11 years) showed no correlation with delayed AGNT (OR 2, 95%CI 0.9–4.6, *P* = 0.1). Patient gender, ethnicity, body weight (z score), and new-onset diabetes mellitus /first DKA episode were also not found to be associated with delayed AGNT. Multivariate analysis adjusted to pH and age on admission showed that glucose above 500 mg/dL on presentation was associated with an increased risk for delayed AGNT, by 3.41-fold (Table 3A). When assessing the association between delayed AGNT and variables such as pH, age, and glucose level on presentation—all as continuous variables (Table 3B), we found that each elevation of serum admission glucose by 1 mg/dL increased the risk for delayed AGNT by 0.4%, (OR 1.004, 95%CI 1,001–1,007) i.e., each increment of 25 mg/dL was associated with a 10% increment of the risk for delayed AGNT (*P* = 0.003). Similar analyses adjusted to age and glucose levels showed that pH below 7.1 at admission was associated with a 2.8-fold risk of delayed AGNT (*p* = 0.059).

**Table 2:** Crude odds ratios of associations of epidemiological and clinical characteristics with delayed AGNT, using logistic regression.

Variable	AGNT ≤ 8 hours	AGNT > 8 hours	Odds ratio	95% CI of Odds ratio	*P* value
pH<7.1 at presentation	10 (32.3%)	21 (51.2%)	3.15	1.2-8.1	**0.015**
History of previous DKA	4 (9%)	8 (16%)	2	0.5-7	0.3
Glucose level at presentation (median, mg/dL)	455.5	563	1.003	1-1.005	**0.01**
Glucose level >500 mg/dL	16 (35.6%)	30 (60%)	2.7	1.2-6.2	**0.02**
Female patient	24 (53.3%)	24 (48%)	0.8	0.4-1.8	0.6
Age (years, mean ± SD)	9.1±4.8	10.8±4.4	1.08	0.99-1.2	0.09
Age >11 years	15 (33%)	25 (50%)	2	0.9-4.6	0.1
Ethnicity (Bedouin origin)	24 (53%)	25 (50%)	0.9	0.4-2	0.7
Weight z score (mean ±SD)	−0.9±3	−0.1±1.2	1.3	0.9-1.9	0.1

AGNT – Anion Gap Normalization Time; CI – Confidence Interval; DKA – Diabetic Ketoacidosis.

**Table 3:** Multivariate analysis adjusted to age, admission pH and glucose. 3A. The three variables are assessed as dichotomous variables.

Parameter	Odds ratio	95% CI	Significance (*P*-value)
Serum glucose on presentation >500 mg/dL	3.410	1.290-9.020	0.013
pH < 7.1	2.607	0.965-7.047	0.059
Age >11 years	1.945	0.727-5.206	0.185

**Table:** 3B. The three variables are assessed as continuous variables.

Parameter	Odds ratio	95% CI	Significance (*P*-value)
Serum glucose on presentation	1.004	1.001-1.007	0.003
pH < 7.1	2.8	1.016-7.798	0.047
Age >11 years	2.275	0.825-6.278	0.112

## Discussion

We found that the median AGNT was 8 h while the median PICU length of stay (LOS) was 23 h. AGNT was found to correlate with lower pH and higher glucose serum levels at presentation. Interestingly, gender, ethnicity, weight, and previous DKA were not found to be associated with delayed AGNT. In our study, pH and bicarbonate serum levels normalization lagged after AG. This observation was previously described (10, 11).

Children in a state of moderate to severe DKA need to be admitted to a specialty unit (PICU or similar) with trained personnel in DKA management, written DKA treatment protocol, and the ability to frequently sample blood tests and respond to any signs of clinical deterioration. Moreover, in case the admitting facility lacks such a unit, it is recommended that the patient be transferred to another healthcare facility by trained medical personnel (3). Specialty beds are a valuable resource and admitting patients beyond the clinical indication may worsen the burden on the healthcare system but may also harm the patient. In this context, clear and simple criteria for DKA recovery and the ability to transfer to a general pediatric floor may improve the management of patient flow and PICU capacity (10).

The general agreement stated that once the acidosis and ketosis were reversed, dehydration was corrected and the child's risk to develop complications decreased—DKA is considered resolved (2, 3). Biochemical criteria for DKA resolution are still under investigation by different groups and are probably also influenced by the institutional ability to perform different laboratory tests such as blood chemistry, blood gas analysis, and serum KB. Serum pH, bicarbonate, AG, and KB were suggested as the preferred markers of resolution alone or in combinations, while urine KB and serum glucose are no longer considered as such (8, 10, 11, 18, 19).

Most recently published DKA treatment guidelines (3) and studies (11, 13) validated the use of venous pH (vpH) for DKA follow-up, management, and resolution. Although we are aware of these publications, we chose to use arterial pH for practical reasons. We often find it challenging to maintain reliable peripheral IV access which will enable hourly blood sampling. And, as hourly blood sticks are unacceptable, using a peripheral arterial line is a good solution in our practice. Arterial line and IV continuous infusion of insulin are not supported outside the PICU. Close laboratory and clinical monitoring should continue as long as the child is receiving these treatments. Once DKA is considered resolved, SC insulin will be prescribed together with the DM diet. At this point, the child will be transferred to the pediatric floor. Current institutional protocol (which is in keeping with published data) enables a patient to be transferred to the floor once pH > 7.30 and bicarbonate >18 mEq\L. In our population, following these guidelines, the median AGNT was 8 h and PICU LOS was 23 h.

Recent studies support our findings. Mrozik et. al (10). followed 59 Australian children with moderate to severe DKA assessed for resolution markers. Resolution criteria were pH > 7.3, HCO3 > 15 mEq\L, AG < 16.1 mEq\L. They found that AG (although higher than our criteria) preceded normalization of pH and bicarbonate by about 7 h in DKA patients with newly diagnosed DM and in severe DKA. They also found hyperchloremic acidosis in over 50% of their patients which was considered to unnecessarily prolong the patient's treatment in the specialty unit.

Von Oettingen et. al (11). explored the utility of vpH, AG, serum bicarbonate, and serum glucose as markers of DKA resolution in children from the northeast USA. Only newly DM diagnosed patients were included. DKA resolution was defined as vpH >7.30, AG equal or below 18 mEq\L. Their conclusions are that serum bicarbonate >15 mEq\L correlates with vpH >7.30 and AG ≤ 18 mEq\L in predicting DKA resolution. The source of the AG reference value of 18 was not mentioned. In their study, the median time to DKA resolution according to AG normalization was 6.7 h (IQR: 5.3–8.8). In moderate and severe DKA, AGNT preceded vpH and bicarbonate normalization in three and six hours respectively.

Rewers et. al (12). in *post hoc* analysis of the PECARN FLUID trial assessed changes in pH, pCO2, glucose, AG, serum electrolytes, and time to metabolic normalization in 714 pediatric patients divided into four treatment groups receiving different fluid protocols. DKA was defined as dextrose >300 mg/dL, pH < 7.20, HCO3 < 15 mEq\L. DKA normalization was determined as pH ≥ 7.32, pCO2 ≥ 38 torr, AG < 12 mEq\L. Fluid infusion rate did not change time to normalization of pH nor pCO2, however, AG was significantly normalized hours earlier in the fast fluid infusion rate group regardless of the fluid type, supporting an earlier finding in adults with DKA.

Trembley et al (9). studied the yield of plasma beta-hydroxybutyrate to define the resolution of DKA. In their study, beta-hydroxybutyrate serum cut-off levels were assessed in comparison to previously published DKA resolution criteria -vpH ≥ 7.3, Serum bicarbonate ≥15 mEq\L and AG ≤ 14 mEq\L. Their analysis showed a robust correlation between beta-hydroxybutyrate levels of less than 1.5 mmol\L and AG of less than 14 mEq\L.

Our study's main outcome measure was AGNT. We focused on AG change over time and show its' normalization precedes pH and bicarbonate normalization. We chose AGNT *a priori* as AG ≤ 12 mEq\L. Following Kamel and Halperin (4), all our patients had a normal range serum albumin which supported our decision to use AG of 12 mEq\L as our cutoff value. AG of 12 mEq\L seems a more conservative measurement when considering DKA resolution. This strongly correlates with KB elimination (level of <1.5 mmol\L) as shown in Trembley et al., results and with improvement in dehydration as shown by Rewers et al. It supports our other hypothesis that AG does not only reflect the resolution of DKA but also may be a good surrogate marker for the recovery of dehydration.

In our study, median pH and bicarbonate normalization following PICU admission showed a gradual increase toward normal levels, while barely reaching or not reaching “normal” physiological levels, even after 30 h, as expected by our institution's DKA resolution criteria. At that point, most patients with DKA have already been transferred to the ward after the resolution of the crisis. pH and bicarbonate levels as sole parameters provide late guidance for treatment de-intensification because their level may be biased by hyperchloremia.

In our cohort, admission pH < 7.1 and glucose levels >500 mg/dL (both markers of severe DKA) were found to be significantly correlated with delayed AGNT. Patient gender, age, ethnicity, and body weight were not found to be associated with AGNT, though these parameters were previously reported to correlate with DKA severity. The small number of children younger than two years prevented us from seeking a correlation between this population and delayed AGNT.

The retrospective design of our study has inherent limitations, including missing data and the inability to assess the patients' hydration state at AGNT, which could have strengthened our findings. Additionally, calculating AG based on retrospective data was challenging due to differences in the timing of blood gas and serum chemistry results recording, requiring correlation between them. However, we mitigated this by excluding patients who did not meet our strict eligibility criteria of less than two hours between blood gas and serum chemistry laboratory results.

Our study highlights the significant difference between the time of pH normalization and AG normalization, suggesting normal AG acidosis secondary to HMA. While our retrospective descriptive study was not designed to elucidate the pathophysiological or biochemical mechanisms underlying the relationship between AG normalization and hyperchloremia, insights can be drawn from previously published studies. For instance, Mrozik et. al (10). found that 58% of DKA patients developed HMA during their hospital stay, while Taylor et al., (13) reported that 94% of patients developed HMA within 20 h of admission to the PICU for DKA treatment. Mrozik et al., also observed a correlation between newly diagnosed cases and high chloride load during treatment, but not with the severity of DKA or admission pH. Palmer et al., (6). reviewed the clinical observation of HMA severity increasing as AG normalizes. HMA is believed to be related to the administration of chloride-rich fluids during DKA treatment, possibly arising from the reabsorption of chloride anions through the kidney's tubules in exchange for KB secretion during DKA recovery. This could explain why chloride serum levels within the normal range during initial DKA treatment tend to rise, as KB is eliminated, and AG normalizes in the serum (6). Importantly, HMA, although not included in the DKA criteria, may mask DKA recovery and unnecessarily prolong PICU admission for DKA patients, as our findings demonstrate. Further research is needed to better understand the underlying mechanisms and implications of HMA in DKA management. One possible approach to better clarify this issue is to conduct a study where DKA patients with normal AG will be divided into two groups. DKA protocol will be discontinued in one group, and the other will stay in DKA protocol until the pH and bicarbonate criteria are met. This future study may help to better evaluate the efficacy of AGNT as a safe and reliable DKA resolution criterion.

Another limitation is the lack of serum KB measurements. Unfortunately, our medical center does not perform these tests routinely. A short survey among Israeli PICUs showed none of them can measure KB routinely during DKA (personal communication).

The study's main strength lies in the non-selective population-based cohort, consisting of data recorded during an entire decade, in a single tertiary medical center. Based on the results of our study that includes a cohort of children with DKA over 10 years, we cautiously suggest that AGNT could be used as DKA resolution criteria and might help to define the right timing to switch from DKA treatment protocol to a less intensive treatment plan. This may facilitate earlier transfer from the specialty unit to the general pediatric floor. We presume that like other studies, for many of our patients with DKA, normal anion gap acidosis, mainly due to hyperchloremic metabolic acidosis, is still perceived as a continuation of the DKA crisis, and patients continue to receive a continuous infusion of insulin, to experience frequent blood sampling and to remain longer than needed in the PICU. Shortening the PICU admission of patients with DKA will surely benefit patients and their families and will enable better utilization of hospital resources. A larger prospective study is needed to better define the role of AGNT in the determination of DKA resolution and treatment de-intensification.

## Data Availability

The datasets presented in this article are not readily available because Based on the Soroka Medical Center, any patient related data can be received upon request from the hospital director's office. Requests to access the datasets should be directed to IL ilazar@bgu.ac.il.
